# Tissue Accumulations of Toxic Aconitum Alkaloids after Short-Term and Long-Term Oral Administrations of Clinically Used *Radix Aconiti Lateralis* Preparations in Rats

**DOI:** 10.3390/toxins11060353

**Published:** 2019-06-18

**Authors:** Xiaoyu Ji, Mengbi Yang, Ka Hang Or, Wan Sze Yim, Zhong Zuo

**Affiliations:** 1 School of Pharmacy, The Chinese University of Hong Kong, Hong Kong SAR, China; SharonChi@link.cuhk.edu.hk (X.J.); yangmengbi@cuhk.edu.hk (M.Y.); 2 School of Chinese Medicine, The Chinese University of Hong Kong, Hong Kong SAR, China; orkh@cuhk.edu.hk (K.H.O.); wendy.yim@cuhk.edu.hk (W.S.Y.)

**Keywords:** *Radix Aconiti Lateralis* preparations, short-term and long-term usage, di-ester diterpenoid alkaloids, mono-ester diterpenoid alkaloids, biodistribution

## Abstract

Although *Radix Aconiti Lateralis* (Fuzi) is an extensively used traditional Chinese medicine with promising therapeutic effects and relatively well-reported toxicities, the related toxic aconitum alkaloid concentrations in major organs after its short-term and long-term intake during clinical practice are still not known. To give a comprehensive understanding of Fuzi-induced toxicities, current study is proposed aiming to investigate the biodistribution of the six toxic alkaloids in Fuzi, namely Aconitine (AC), Hypaconitine (HA), Mesaconitine (MA), Benzoylaconine (BAC), Benzoylhypaconine (BHA) and Benzoylmesaconine (BMA), after its oral administrations at clinically relevant dosing regimen. A ultra-performance liquid chromatography-tandem mass spectrometry (UPLC–MS/MS) method was developed and validated for simultaneous quantification of six toxic alkaloids in plasma, urine and major organs of Sprague Dawley rats after oral administrations of two commonly used Fuzi preparations, namely Heishunpian and Paofupian, at their clinically relevant dose for single and 15-days. Among the studied toxic alkaloids and organs, BMA demonstrated the highest concentrations in all studied organs with liver containing the highest amount of the studied alkaloids, indicating their potential hepatotoxicity. Moreover, tissue accumulation of toxic alkaloids after multiple dose was observed, suggesting the needs for dose adjustment and more attention to the toxicities induced by chronic use of Fuzi in patients.

## 1. Introduction

The processed lateral root of *Radix Aconiti Lateralis* is known as Fuzi, an extensively used traditional Chinese medicine in China and other Asian countries [1]. Fuzi is recognized as a treatment for cardiovascular diseases, rheumatism arthritis, bronchitis, pains and hypothyroidism, etc. [1,2]. Among the 500 well-known traditional Chinese medicine (TCM) formulae used in clinics, approximately 13.2% include Fuzi [1]. In clinical practice, Fuzi-containing prescriptions are usually given orally, with 81.8% of them used at 5 g/person/day, 9.1% of them used at 10 g/person/day and the rest used at 15 g/person/day [3].

Although Fuzi has promising therapeutic effects, aconitum poisoning has been extensively reported with 17 cases from Taiwan during 1990–99, 39 cases form Hong Kong during 2012–19, 2017 cases from mainland of China during 1989–2008, and 121 cases from Korea during 1995–2007 [4,5]. In clinical poisoning cases, the patients usually had acute onset of illness within half an hour to several hours after consumption of Fuzi with cardiotoxic and neurotoxic effects as the main outcomes. Cardiotoxicity symptoms included palpitation, arrhythmia with slow heart rate, and low blood pressure. Neurotoxicity features included numbness in the oral cavity, tongue, face, extremities and body, muscle weakness and dizziness. Arrhythmia and muscle weakness-induced breathing difficulties may lead to death in severe cases [6].

**Figure 1:**
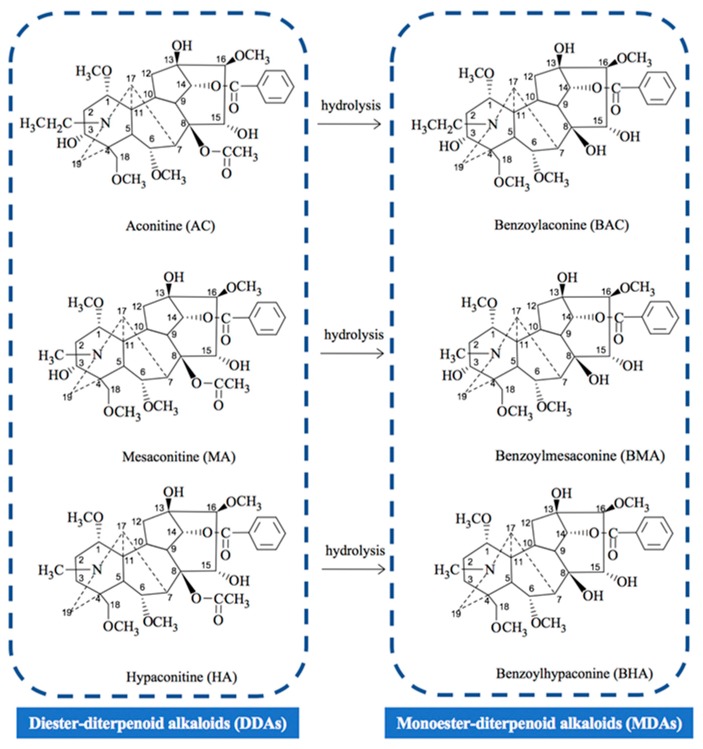
Relationships between the studied di-ester- and mono-ester- diterpenoid alkaloids of Fuzi.

The principal toxic ingredients in Fuzi are C_19_-diterpenoid alkaloids, including three di-ester diterpenoid alkaloids (DDAs) and three mono-ester diterpenoid alkaloids (MDAs) [1,3]. As shown in Figure 1, processing of Fuzi would make DDAs lose their acetyl group at C_8_ and become MDAs. Based on their LD_50_ values on mice, the toxicities of MDAs were 1/700–1/100 of that of DDAs [7]. MDAs could subsequently lose the benzoyl ester group at C_14_ to generate non-toxic non-ester type alkaloids (NDAs) [7,8,9,10,11].

Previous mechanistic studies on Fuzi-induced toxicity mainly focused on the cardiotoxicity of the DDAs. The DDA-induced arrhythmogenic effects, which have been frequently observed after ingestion of Fuzi, were well recognized to be caused by activating the voltage-dependent sodium channel on the cell membranes in excitable tissues such as myocardium [1,12,13,14]. More recently, other cardiotoxic mechanisms for toxic aconitum alkaloids were proposed. Aconitine was found to be able to perturb intracellular calcium homeostasis via Na^+^-Ca^2+^ exchange system [1] and sarco/endoplasmic reticulum Ca^2+^-ATPase [12], leading to further cell apoptosis in heart [12]. Therefore, damaged intracellular calcium homeostasis was considered as the key cause of ventricular arrhythmias of aconitine [15]. In addition to heart, aconitine induced apoptosis was found in other organs including liver [16], brain [17], and kidney [18].

Based on various processing methods used by Chinese medicine practitioners, there are ten types of Fuzi preparations [19,20,21], among which Heishunpian and Paofupian are the most common ones and serve as the major components in classical Chinese medicine formulae Sini Tang for acute treatment (usually single dose) and Fuzi Lizhong Tang for sub-chronic treatment (could be as long as two weeks), respectively. After excavation, crude Fuzi was boiled in hot water, roasted with salts and finally stained into black slice to make Heishunpian, which could be further roasted with sands until inflated and slightly discolored to form Paofupian [22]. It was reported that some poisoning cases occurred after consumption of Heishunpian even at doses within its therapeutic range of 3–15 g/person/day [23]. Although compared with Heishunpian, further hydrolysis processing procedure for Paofupian is expected to lower its toxic alkaloid contents (especially for DDAs), the toxicities of Paofupian should not be overlooked considering its wider indications and longer period of intake.

The concentrations of the toxic alkaloids DDAs in plasma and cardiac tissue have been found positively correlated with the cardiac toxicity after single dose of Fuzi in mice and rats [24], suggesting that plasma concentrations of DDAs could be applied to monitor cardiac toxicities of Fuzi in clinical practice. However, the relationship between plasma/tissue concentration of toxic alkaloids and the other potential organ toxicity of Fuzi is largely unknown. Previous studies depicted in vivo biodistribution of toxic alkaloids after single dose of Fuzi, while there is a lack of information on the biodistribution profiles of toxic alkaloids after multiple dose of Fuzi preparation, leading to no established toxicokinetic-toxicity correlation for long-term use of Fuzi. In addition, most of the previous biodistribution studies [25,26] on Fuzi used their own Fuzi extract, and the effect of Fuzi preparations with different processing method on biodistribution profiles of toxic alkaloids remains unclear. It is hard to generate any conclusion without standardizing the Fuzi preparations used in the studies.

To give a more complete understanding of the biodistribution profiles of toxic alkaloids to see how they relate to Fuzi-induced toxicities, the current study was conducted to establish an LC/MS/MS method for simultaneous determination of six toxic aconitum alkaloids in rat plasma, urine and major organs, followed by its application to investigate their biodistributions after single and 15-day oral administrations of Heishunpian and Paofupian at their clinical dosing regimen in rats.

## 2. Results

### 2.1. LC/MS/MS Method Development and Validation for Simultaneous Determination of Six Toxic Aconitum Alkaloids in Different Biological Matrix

#### 2.1.1. Optimization of Chromatographic and Mass Conditions

**Figure 2:**
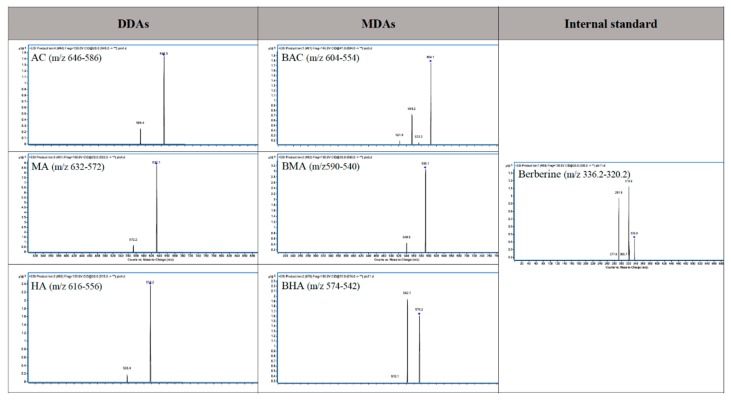
Product ion spectra of AC, MA, HA, BCA, BHA, BMA, and Berberine (IS).

The optimal parameters including precursor ion, product ion, fragmentor and collision energy are listed in Table 1. Under such optimized MS/MS condition, the response for each analyte was high enough for detection in biological matrix. Spectra of product ion scan on six targeted toxic alkaloids are shown in Figure 2.

#### 2.1.2. Optimization of Solid Phase Extraction (SPE) Conditions for Sample Treatment

Solid phase extraction (SPE) using a mixed-mode cation-exchange (MCX) cartridge was adopted to separate the targeted toxic alkaloids from the biological matrix in brain and liver homogenates. According to previous published studies [27], 5% ammonium hydroxide in 70% methanol was used to elute compounds binding on the MCX cartridge. However, by adopting such elution method, we found the response of targeted alkaloids were not high enough for detection, due to the extremely low recoveries of Hypaconitine (HA) and Benzoylmesaconine (BHA). Under such elution condition, the recovery of Aconitine (AC), Mesaconitine (MA), HA, Benzoylaconine (BAC), BMA and Benzoylhypaconine (BHA) were 75%, 86%, 9%, 39%, 48% and 0%, respectively. Therefore, a series of eluent with different percentage of methanol ranging from 15% to 95% were tested further (data not shown). The results revealed that the best recoveries were achieved by 5% ammonium hydroxide in 95% methanol with over 93% recoveries for all six analytes. Based on such optimization, 5% ammonium hydroxide in 95% methanol was selected out as final eluent for solid phase extraction.

#### 2.1.3. Method Validation

As demonstrated in Table A1, the optimized liquid chromatography tandem-mass spectrometry (LC-MS/MS) conditions for the six toxic alkaloids could provide satisfactory linearities (r^2^ ≥ 0.99) in plasma (0.5–100 ng/mL), urine (0.5–200 ng/mL) and different organs (range from 0.5 to 200 ng/mL). Lower Limit of Quantification (LLOQs) in plasma was 0.5 ng/mL, while no higher than 2 ng/mL in urine and different organs. The results for intra- and inter-day accuracy and precision are shown in Table A2, Table A3, Table A4, Table A5, Table A6 and Table A7. The accuracy and precision of the plasma and organs assays at low, medium and high concentrations of targeted alkaloids were within ±15% bias and 15% Relative Standard Deviation (RSD), which met the criteria set in the guidance issued by U.S. Food and Drug Administration (FDA) [28]. The extraction recoveries in biological matrices remained consistent.

The stability results were also shown in Table A1, Table A2, Table A3, Table A4, Table A5, Table A6 and Table A7. According to the results, alkaloids concentration detected in stability samples were within 15% of nominal concentrations after three freeze-thaw cycles, 4 h on bench top, 12 h in the auto-sampler of LC/MS/MS system, and 30 days at −80 °C.

### 2.2. Content of Six Toxic Aconitum Alkaloids in Studied Radix Aconiti Lateralis Preparation

**Figure 3:**
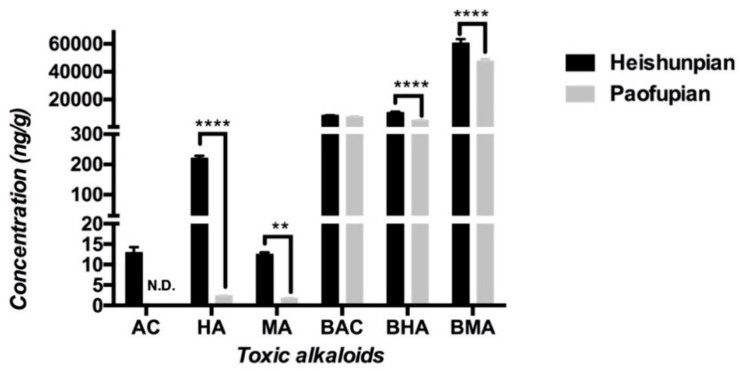
Concentrations of AC, HA, MA, BAC, BHA, and BMA in the Heishunpian and Paofupian crude herb (*n* = 3, ** *p* < 0.01, **** *p* < 0.0001, N.D.: not detectable, below the listed LLOQ of each rat tissue in Table A1).

Contents of the six toxic alkaloids in Heishunpian and Paofupian are depicted in Figure 3. It was found that the content of BMA was the highest among all six toxic alkaloids and the content of HA was the highest among the studied three DDAs. It was also noted that total contents of MDAs were much higher than those of DDAs in both Fuzi preparations with the contents of MDAs 50–5000 times of DDAs in Heishunpian and 2000–30,000 folds of DDAs in Paofupian. Comparing the toxic alkaloids contents in two Fuzi preparations, higher contents of toxic alkaloids were detected in Heishunpian than that in Paofupian, especially for HA, MA, BHA and BMA. The content of AC was 12.94 ng/g in Heishunpian, while it was not detectable in Paofupian.

Based on the contents of six toxic alkaloids in two Fuzi preparations, the toxic alkaloid dosages of 30 g/kg Heishunpian in rat were: BMA (1814 μg/kg), BHA (321 μg/kg), BAC (254 μg/kg), HA (6.61 μg/kg), AC (0.39 μg/kg) and MA (0.38 μg/kg), and the toxic alkaloid dosages of 30 g/kg Paofupian were: BMA (1430 μg/kg), BAC (227 μg/kg), BHA (150 μg/kg), HA (0.07 μg/kg), MA (0.05 μg/kg) with no detectable AC.

### 2.3. Biodistributions of Toxic Aconitum Alkaloids after Oral Administrations of the Studied Radix Aconiti Lateralis Preparations

**Figure 4:**
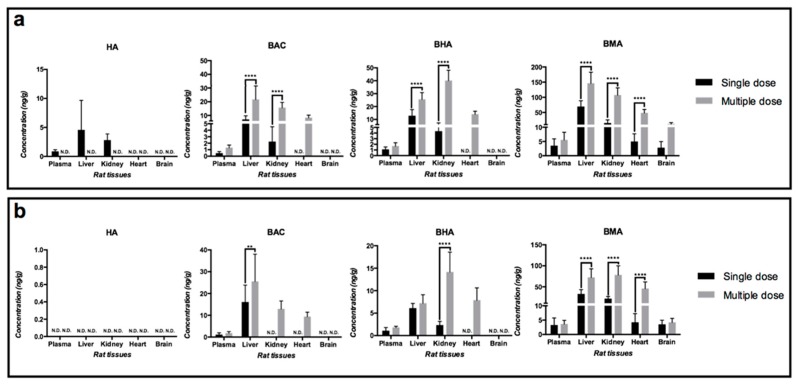
Profiles of HA, BAC, BHA, and BMA in rat plasma and different organs at 2 h after both bolus dose and 15-day consecutively dose of (**a**) Heishunpian and (**b**) Paofupian at 30 g/kg to rats (*n* = 8, ** *p* < 0.01, **** *p* < 0.0001, N.D.: not detected, below the listed LLOQ of each rat tissue in Table A1).

Toxic alkaloid concentrations in rat tissues after oral administrations of 30 g/kg Heishunpian and 30 g/kg Paofupian both for single dose and multiple dose to rats are illustrated respectively in Figure 4. The contents of AC and MA in all the tested tissues of all treatment groups were below their detection limits and therefore are not shown in Figure 4. 

Consistent with their contents in the dosed Fuzi preparations (Figure 3), the contents of DDAs in all the tested tissues are lower than those of MDAs (Figure 4a,b). Three types of MDAs could be detected in the majority of the organs regardless giving either single dose or multiple dose of both two Fuzi preparations (Figure 4a,b). The biodistribution levels of three MDAs into rat tissues followed the order of BMA >> BHA ≈ BAC (Figure 4a,b). In terms of DDAs, HA was the only detectable one after single dose of Heishunpian (Figure 4a), while no DDA can be detected after single or multiple dose of Paofupian due to the extremely low contents of DDAs in the administrated Paofupian (Figure 3).

Among all the studied organs, liver contained highest amount of all detectable toxic alkaloids. Closely following liver, kidney contained relative high concentration of toxic alkaloids. Only once exception occurred in biodistribution of BHA after multiple dose of Paofupian, where concentration of BHA in kidney went beyond that in liver.

In addition to plasma and organs, concentration of toxic alkaloids in urine had also been tested. Althought no difference was shown after single or multiple dose of Paofupian, after oral administration of Heishunpian, more numbers of studied toxic alkaloids could be detected in urine sample (all toxic alkaloids for single dose, four toxic alkaloids for multiple dose) than that in plasma sample (four toxic alkaloids for single dose, three toxic alkaloids for multiple dose). Furthermore, concentrations of toxic alkaloids in urine were 10–80 times higher than those in plasma after dosing Heishunpian or Paofupian to rats.

Tissue accumulation of MDAs due to long-term treatment–giving Heishunpian or Paofupian preparation once daily for consecutive 15 days–was clearly observed (Figure 4a,b). Compared with single dose, multiple dose of Heishunpian resulted in higher concentrations of MDAs in liver, kidney and heart (Figure 4a). Similarly, multiple dose of Paofupian resulted in more content of BAC in liver or BHA in kidney, and BMA in four major organs except brain (Figure 4a). Considering the only variable behind such comparison was treatment duration, it was reasonable that attributing higher content of toxic alkaloids in rat tissues to longer treatment duration, or more dosing times in other words.

### 2.4. Comparison of Dose-Normalized Toxic Aconitum Alkaloid Contents in Rat Plasma, Urine and Major Organs

Since the doses of each toxic alkaloids in two Fuzi preparations varied, the contents of toxic alkaloids in each organ were normalized by their individual doses as shown in Table 2 (single dose) and Table 3 (multiple dose). The detected DDAs had higher dose-normalized contents than MDAs in plasma, urine, and major organs including liver, kidney and heart, which was more evident in rats after giving single dose of Heishunpian. In addition, the dose-normalized contents of all detectable toxic alkaloids in different rat organs were in the order of liver > kidney ≈ heart > brain.

Dose-normalized toxic alkaloid contents in each organ were adopted for comparison of the biodistribution of toxic alkaloids between two different Fuzi preparations, Heishunpian and Paofupian. It could be summarized from Table 2 that the dose-normalized total toxic alkaloids contents in rat tissues (plasma, urine, liver, kidney and heart) from single dose of Heishunpian treated group were significantly higher than that from single dose of Paofupian treated group. Table 3, where gave the dose-normalized content of toxic alkaloids after multiple dosing of Fuzi preparations, demonstrated that compared with Paofupian, toxic alkaloids from Heishunpian could more easily distribute to rat liver, kidney, brain as well as more possibly be detected in rat urine preparation.

## 3. Discussion

Existing studies mainly focused on cardiac toxicities induced by DDAs from Fuzi. Mechanisms behind DDAs-induced arrhythmogenic effects have been well elaborated [1,12,13,14,15], and the relationship between DDAs concentrations in heart tissue and cardiac toxicity has been established [24]. However, only few studies have mentioned the hepatic and renal toxicities of Fuzi [16,18]. Furthermore, there has been a lack of information on concentrations of major toxic alkaloids in liver and kidney after both short-term and long-term intake of Fuzi preparations. Therefore, the current study for the first time investigated the biodistributions of the six DDAs and MDAs from commonly used processed Fuzi preparations after both single and multiple oral administrations in rats at the their clinically relevant doses. Such preclinical tissue distribution profiles were expected to provide us with better understanding of in vivo distribution behaviors of these alkaloids for more in-depth investigation of their toxicities and safer use of Fuzi. The current LC/MS/MS analytical method achieved simultaneously detection of all six toxic aconitum alkaloids across urine, plasma, and four major organs, providing a wider range of application with higher efficiency than previous analytical methods which were applied to detect fewer types of toxic alkaloids in urine or plasma only [29,30].

Different contents of six alkaloids in the two studied Fuzi preparations were highly related to different processing procedure of Heishunpian and Paofupian. Paofupian had one more step of roasting with sands to do from Heishunpian. Long time high temperature condition facilitated the hydrolyzation of DDAs and MDAs to MDAs and NDAs [10]. As a result, there were less DDAs and MDAs in Paofupian, explaining why traditional Chinese medicine prefer Paofupian for long-term treatment. Percentages of DDAs in Heishunpian and Paofupian were determined to be 2.46 × 10^−5^%, and 4.11 × 10^−7^% respectively. The DDA contents in both two preparations were much less than 0.01%—the criteria listed in 2015 Chinese Pharmacopoeia [31]. Therefore, the tested Heishunpian and Paofupian met the criteria in 2015 Chinese Pharmacopoeia. In contrary to the extremely low contents of DDAs, the contents of MDAs were 300 and 15,000 folds higher in Heishunpinan and Paofupian respectively, leading to relatively high dose of the toxic MDAs in this two Fuzi preparation.

Our biodistribution data for the first time revealed the effect of long-term dose of Fuzi preparations (both Heishunpian and Paofupian) on the biodistribution profiles of toxic alkaloids. It was found that long-term exposure of Fuzi preparation could lead to significant accumulation of MDAs in liver, kidney and heart, while no accumulation was found for DDAs. Since the phase I and phase II metabolic rate of MDAs in human liver microsome are less than 10% [32], the accumulation of MDAs in the major organs may not be related to altered metabolic enzyme activities. On the other hand, altered activities of transporters [33] and impaired organ functions might be the reasons responsible for such accumulation, which warrants further investigation on the underlining mechanism. Our novel findings on the accumulation of MDAs in liver, kidney and heart after ingestion of Fuzi preparations suggested higher risk of organ injury when Fuzi was used for sub-chronic treatment over 15 days.

As shown by the contents of toxic alkaloids in the two Fuzi preparations (Figure 3), the doses of MDAs were 300–15,000 folds higher than those of DDAs. As a result of the high doses, MDAs demonstrated the remarkably higher amounts in plasma, urine and major organs regardless of the treatment duration compared with DDAs. Based on the reported LD_50_ for a single oral dose of MDAs in mice (0.81 g/kg for BMA, 1.50 g/kg for BAC, and 0.83 g/kg for BHA [7]), MDAs were also toxic and lethal. The high in vivo exposure and the marked cumulation of MDAs in organs suggested the toxicity of MDAs warranted further attention and investigation.

In addition, our findings also revealed that the biodistributions of six toxic alkaloids from the two Fuzi preparations still differed from each other even after dose normalization, (Table 2 and Table 3). Such difference between Heishunpian and Paofupian was highly possibly due to composition and relative ratio changes of co-occurring components existing in Heishunpian and Paofupian. Different processing procedures and substances introduced during decoction and roasting, such as sand and salt, may give further explanation of co-occurring component’s source in Heishunpian and Paofupian.

It was also noted that the dose-normalized contents of DDAs were higher than those of MDAs in majority of studied organs (Table 2 and Table 3). One of the explanations for such phenomena could be the higher lipophilicities of DDAs compared with that of MDAs (calculated LogP by ALOGPS 2.1 software of studied six toxic alkaloids are 1.68 for AC vs 1.09 for BAC, 2.07 for HA vs 1.35 for BHA, and 1.37 for MA vs 0.69 for BMA,), which may potentially result in higher membrane permeability, higher tissue binding affinity and less excretion. Moreover, the toxicities of DDAs was around 1000-fold stronger than MDAs, according to their LD_50_ values [7]. Therefore, due to their higher recoveries in organs and more potent toxicity compared with MDAs, the quality control on the contents of DDAs was essential to avoid Fuzi-induced toxicity. Apart from quality control, sufficient decoction time of Fuzi crude herb which allowed effective hydrolysis of DDAs to MDAs was necessary.

Our findings on higher possibility of toxic alkaloids occurrence in urine sample when compared to plasma sample provided a reasonable explanation of a previous clinical poisoning case [1] reported that concentration of toxic alkaloids in urine was much higher than those in blood and supported urine test for Fuzi poisoning diagnosis in clinic.

Last but not the least, current findings confirmed that the toxic alkaloid levels were remarkably high in the liver and kidney, relatively low in the heart and blood with only a trace amount recovered in the cerebrum, consistent with previous biodistribution result in clinical poisoning cases [34] and preclinical studies [25,26]. However, it should be emphasized that they gave extremely high dose of Fuzi extract ranging from 0.2–10 g/kg [35,36] or pure toxic alkaloid such as AC or HA ranging from 0.1–2 mg/kg [37,38] to rats. Differently, 30 g/kg of Fuzi used in our study referred to reported maximum dose in clinics [39]. Different from others using organic solvent to extract Fuzi [40], current study mimicked patient’s intake habit that suspending the granule in hot water and stirring well. Hepatotoxicity and nephrotoxicity caused by Fuzi have been previously evidenced by upregulated cell apoptosis factor [16], dose-dependent edema in liver tissue, high level expression of Alanine Aminotransferase (ALT), Aminotransferase (AST) and Lactate Dehydrogenase (LDH) in serum [41] after oral administrations of Fuzi extract or pure toxic alkaloid compounds. Current results gave a warning that hepatotoxicity and nephrotoxicity caused by Fuzi preparations—no matter if taken at one time or multiple times—are worthy of closer attention in clinical practice. Our current study revealed the remarkably high tendency of the toxic alkaloids to distribute into the liver after oral ingestion of Fuzi at a clinically relevant dosing regimen, which could partially explain the hepatotoxicity reported in previously conducted studies.

## 4. Conclusions

The current developed and optimized LC/MS/MS method for quantitative determination of the six toxic aconitum alkaloids was specific and sensitive for biodistribution study in rats. Among the six toxic alkaloids, BMA had the highest content in the investigated Fuzi preparation, which also led to its highest content in rat tissues.

Our current study for the first time demonstrated not only the remarkably high tendency of toxic aconitum alkaloids distributing into the liver and kidney, but also their significant accumulation in major organs after long-term oral administrations of the Heishunpian and Paofupian concentrated granules at clinically relevant dosage to rats. Therefore, clinical use of Fuzi in patients with sub-chronic and even chronic disease may need more precise adjustment on dosage and more attention to liver injury induced by Fuzi. Additionally, our study revealed that co-occurring components existing in Heishunpian increase the in vivo exposure and tissue content of six toxic aconitum alkaloids existing in Fuzi.

## 5. Materials and Methods 

### 5.1. Materials, Reagents and Animals

All the reagents used in the current study were at least of analytical grade. Formic acid was supplied by BDH Laboratory Supplied Ltd. (Kampala, Ukraine). Acetonitrile and methanol were purchased from RCI Laboscan Ltd. (Bangkok, Thailand) and both of them were HPLC-grade. Deionized water was used for the preparation of all solutions. Six toxic aconitum alkaloids, including aconitine, hypaconitine, mesaconitine, benzoylaconine, benzoylhypaconine and benzoylmesaconine, and Berberine hydrochloride which was used as internal standard (IS) were all purchased from Sigma-Aldrich (St. Louis, MO, USA). Heishunpian or Paofupian concentrated granule were obtained from Purapharm Co., Ltd. (Hong Kong SAR, P. R. China, Batch Number A1601163 for Heishunpian, A1701434 for Paofupian).

Adult male Sprague Dawley rats (weighing 200–220 g) were supplied by the Laboratory Animal Services Centre, the Chinese University of Hong Kong, HKSAR, China. This study was approved on 9 February 2018 by the Animal Experimentation Ethics Committee of the university (Reference No. 17/219/HMF-5-B).

### 5.2. Chromatographic and Mass Spectrometric Conditions

Separation and quantification of DDAs including Aconitine (AC), Hypaconitine (HA), Mesaconitine (MA), MDAs including Benzoylaconine (BAC), Benzoylhypaconine (BHA), Benzoylmesaconine (BMA) and internal standard (Berberine) were performed on Agilent 1290 Ultrahigh performance liquid chromatograph coupled to an Agilent 6430 Triple Quad LC/MS (UPLC–MS/MS) with electrospray ionization (ESI) (Agilent Technologies Inc., Santa Clara, CA, USA). A Waters Acquity UPLC BEH C18 column (2.1 × 50 mm, 1.7 μm, Waters Corporation, Milford, MA, USA) was used for chromatographic separation. The mobile phase was composed of an aqueous solution of 0.1% formic acid (Solvent A) and acetonitrile containing 0.1% formic acid (Solvent B). The gradient profile was as follows: 0–4 min with a linear gradient of B from 10 to 45 % and 4–8 min with a linear gradient of B from 45 to 70%, for 8–9 min the composition of B is maintained at 70%, finally the column was re-equilibrated. The flow rate was 0.15 mL/min. The injection volume was 10 μL. The entire eluent was ionized via an ESI source operating in the positive mode and monitored by MS/MS detection in the multiple reaction monitoring (MRM) mode. The optimization of the MS/MS conditions were conducted by directly injecting the individual analyte solutions in methanol at a concentration of 500 ng/ml with a mobile phase composition and flow rate equivalent to those at the time the analyte would elute from the UPLC column.

### 5.3. Preparation of Standard Solution and Quality Control Samples

Tested alkaloid (1 mg) was dissolved in 1 mL 50% methanol in water (*v/v*, containing 2.5% formic acid) to obtain a stock solution (1 mg/mL). The stock solutions of each tested alkaloid were then mixed accordingly to obtain a stock solution containing 1 mg/mL of each toxic alkaloid. 

The stock solutions with mixed alkaloids were diluted with 50 % methanol in water (*v/v*, containing 2.5% formic acid, for quality control (QC) samples in granule content determination) or deionized water (for QC samples in rat tissue content detection to a series of concentration level ranging from 0.5–1000 ng/mL). Detailed concentration levels depended on sample content in various matrix to be tested as shown in Table A2, Table A3, Table A4, Table A5, Table A6 and Table A7.

### 5.4. Sample Preparations

All the collected organs were homogenized with two volumes normal saline containing 1 mmol/L hydrochloric acid to obtain the relevant homogenate.

After centrifuging blood at 8000 rpm for 3 min, 300 μL plasma was obtained for further protein precipitation with 600 μL acetonitrile according previous description [42]. In addition, collected urine sample (300 μL) and organ homogenate from kidney (300 μL) and heart (300 μL) were also subject to precipitate protein with 600 μL acetonitrile. All the above mixtures were centrifuged at 13,000 rpm for 10 min to obtain the supernatant.

For liver and brain sample preparations, 300 μL of their homogenate was loaded to MCX cartridge (Oasis^®^ Part Number 186000252). After washing the cartridge with 1 mL of 0.1 M HCl in H_2_O followed by 1 mL of methanol, the analytes were eluted with 750 μL methanol containing 5% ammonium hydroxide.

All the supernatant or eluent resulted from above sample treatment experienced dryness under a stream of nitrogen gas. The residues were reconstituted with 100 μL 50% methanol containing 2.5% formic acid with 10 μL of which injected to LC/MS/MS for analyses of the six toxic alkaloids.

### 5.5. LC/MS/MS Method Validation 

The method developed was validated based on the guidelines provided by U.S. Food and Drug Administration (FDA) [28], including specificity, linearity, lower limit of quantification (LLOQ), accuracy, precision, recovery, matrix effect, and stability.

#### 5.5.1. Specificity

The specificity of the method was conducted by comparing the chromatographs of blank rat plasma, urine and organ homogenate samples with that of blank rat samples spiked with standard solutions and rat plasma/brain homogenate after oral administration of Heishunpian or Paofupian.

#### 5.5.2. Linearity and Sensitivity

Calibration samples were prepared as described in Section 5.3. The calibration curves were obtained by plotting the ratio of peak area of six toxic alkaloids to IS against the concentration of six toxic alkaloids. The coefficient of determination (r^2^) was calculated, which with a value greater than 0.99 was considered as an indicator of good linearity. The lower limit of quantification (LLOQ) was defined as the lowest concentration of the calibration curve with a signal-to-noise (S/N) peak ratio greater than 5:1.

#### 5.5.3. Accuracy and Precision

The intra-day accuracy and precision test were conducted by analyzing QC samples at nominal concentrations (described in Table A2, Table A3, Table A4, Table A5, Table A6 and Table A7) with at least 5 replicates for each concentration within one day. The inter-day accuracy and precision were determined on three days separately. Accuracy and precision should be within ± 15% bias and 15% RSD respectively.

#### 5.5.4. Recovery and Matrix Effect

The recovery of extraction was determined by the peak area of six toxic alkaloids spiked with the biological matrices followed by extraction against the peak area of six toxic alkaloids spiked to the extracted biological matrices. The matrix effects caused by different biological matrix including plasma, urine, liver, kidney, brain and heart homogenate were calculated by comparing the standard curve in above biomatrix with that in 50% methanol in water (*v/v*, containing 2.5% formic acid). Recoveries and matrix effects of six toxic alkaloids at the levels of Limit of Quantification (LOQs), Middle of Quantification (MOQs) and High of Quantification (HOQs) should be consistent and reproducible.

#### 5.5.5. Stability

Freeze-thaw stability test was conducted by putting the QC samples (prepared in Section 5.3) to three cycles of freeze (−80 °C) thaw (25 °C) before extraction. The stability of six toxic alkaloids at bench top and in the auto-sampler were conducted after sample extraction on the bench (25 °C) for 4 h or in the auto-sampler (8 °C) for 12 h respectively. Long-term stability of six toxic alkaloids was tested after keeping sample at −80 °C for 30 days.

### 5.6. Application of the Developed LC/MS/MS Method for Biodistribution Study of Radix Aconiti Lateralis Preparations in Rats

#### 5.6.1. Quality Control of the Studied Radix Aconiti Lateralis Preparations

Heishunpian or Paofupian concentrated granules are most frequently prescribed Fuzi processing products in local clinics. Based on content analyses of DDAs in Fuzi processed products in 2015 edition Chinese Pharmacopoeia [29] and the concentrated ratio of current Heishunpian or Paofupian granules to its processed herb equals (1:5), the contents of DDAs in the granules were analyzed as follows to compare with the criteria in 2015 Chinese Pharmacopoeia.

About 5 g concentrated granules were firstly suspended in 10 mL 50% Methanol which containing 2.5% formic acid, then sonicated at 25 °C for 30 min. Filtrating through 0.45 μm polypropylene filter, filtrate was divided into two part. One of filtrate (200 μL) was mixed with 200 μL 20 ng/mL internal standard then injected into LC/MS/MS spectrometry, focusing on DDAs quantification. The other filtrate need be 20 times diluted before mixing with internal standard, focusing on MDAs detection. The content of DDAs come from per gram Fuzi processed herb will be indicated as DDAs percentage (%) and calculated by following Equations (1–2) for its comparison with those criteria for Fuzi processed product:

(1) $$\mathrm{DDAs}\;\mathrm{percentage}\;\left(\%\right)=\frac{\mathrm{Content}\;\mathrm{of}\;\mathrm{DDAs}\;\mathrm{in}\;\mathrm{per}\;\mathrm{gram}\;\mathrm{concentrated}\;\mathrm{granule}}{\mathrm{Concentrated}\;\mathrm{ratio}}\times 100\%$$

(2) Concentrated ratio=5

#### 5.6.2. Animal Studies

Commercially available concentrated granules of Heishunpian and Paofupian were orally given to Sprague Dawley rats (*n* = 8 for each group) at a bolus of 6 g/kg (equivalent to 30 g/kg crude herb) for both one time and once daily lasting 15 days.

To mimic clinical use of Fuzi preparations, 5 g concentrated granule of Heishunpian and Paofupian (Batch number A1601163 and A1701434 respectively) were suspended in 10 mL boiling water followed by sonication until evenly suspense in water. Since reported cardiotoxicity usually happened at 1–2 h post-dosing [2,14], two hours after the single dose of Fuzi preparations or two hours after the oral administration of Fuzi preparations on the last day for multiple dosing, rats were sacrificed followed by collection of blood and organs including liver, kidney, heart, and brain after cardiac perfusion with 150–200 mL saline. For single dose of Heishunpian or Paofupian, urine from each rat was collected using metabolic cage during the period from dosing to sacrificing. For multiple dose of above Fuzi preparations, urine from each rat was collected using metabolic cage during the period from dosing on Day 15 to sacrificing. All collected samples were treated as described in Section 5.4 and analyzed by the developed and validated LC/MS/MS method shown in Section 5.2.

### 5.7. Data Analyses

Toxic alkaloid contents were expressed as the mean ± standard deviation. To give further explanation to biodistribution results, dose-normalized content of toxic alkaloid in each organ is calculated using following Equations (3)–(5). For Paofupian treated group, the dose on last day was served as dosed alkaloid content to calculate dose-normalized content of toxic alkaloid in each tissue.

Partition coefficient parameter (LogP) of studied six toxic alkaloids from Fuzi was calculated using ALOGPS 2.1 software.

(3) $$\mathrm{Dose}\;\mathrm{normalized}\;\mathrm{content}\;\mathrm{of}\;\mathrm{toxic}\;\mathrm{alkaloid}=\frac{\mathrm{Detected}\;\mathrm{Alkaloid}\;\mathrm{content}}{\mathrm{Dosed}\;\mathrm{Alkaloid}\;\mathrm{content}}$$

Detected Alkaloid content (ng) = detected Alkaloid conc.(ng/g) × tissue weight (g)(4)

Dosed Alkaloid content (ng) = granule alkaloid conc.(ng/g) × dosed granule content (g)(5)

## Figures and Tables

**Table 1:** List of selected Multiple Reaction Monitoring (MRM) parameters, fragmentor, and collision energy for studied analytes and internal standard (IS).

Compound	Precursor Ion (m/z)	Product Ion (m/z)	Fragmentor (mV)	Collision Energy (mV)
AC	646	586	130	25
MA	632	572	150	37
HA	616	556	125	33
BAC	604	554	145	41
BMA	590	540	130	33
BHA	574	542	185	37
Berberine (IS)	336.2	320.2	130	25

**Table 2:** Comparison of dose-normalized toxic alkaloids content (× 10^−3^) in rat plasma, urine and different organs at 2 h post-dosing after single dose of 30 g/kg Heishunpian and single dose of 30 g/kg Paofupian to rats. (*n* = 8).

Tissue	Liver		Kidney		Heart		Brain		Plasma		Urine	
Compound	Heishunpian	Paofupian	Heishunpian	Paofupian	Heishunpian	Paofupian	Heishunpian	Paofupian	Heishunpian	Paofupian	Heishunpian	Paofupian
AC	N.D.	N.D.	N.D.	N.D.	N.D.	N.D.	N.D.	N.D.	N.D.	N.D.	N.D.	N.D.
HA	10.89 ± 11.11	N.D.	1.38 ± 0.47	N.D.	0.09 ± 0.14	N.D.	N.D.	N.D.	3.91 ± 1.49	N.D.	17.10 ± 13.51	N.D.
MA	N.D.	N.D.	N.D.	N.D.	N.D.	N.D.	N.D.	N.D.	N.D.	N.D.	9.63 ± 6.93	N.D.
Total DDAs	10.89 ± 11.11	N.D.	1.38 ± 0.47	N.D.	0.09 ± 0.14	N.D.	N.D.	N.D.	3.91 ± 1.49	N.D.	26.73 ± 19.88	N.D.
BAC	0.44 ± 0.13	2.84 ± 0.12	0.03 ± 0.03	N.D.	N.D.	N.D.	N.D.	N.D.	0.05 ± 0.03	0.04 ± 0.00	0.25 ± 0.19	0.11 ± 0.00
BHA	0.66 ± 0.25	1.37 ± 0.06	0.04 ± 0.03	0.10 ± 0.00	0.01 ± 0.00	N.D.	0.01 ± 0.00	N.D.	0.10 ± 0.05	0.06 ± 0.00	0.28 ± 0.17	0.07 ± 0.00
BMA	0.64 ± 0.21	0.82 ± 0.04	0.03 ± 0.02	0.11 ± 0.00	N.D.	0.00 ± 0.00	N.D.	0.02 ± 0.00	0.06 ± 0.04	0.02 ± 0.00	0.31 ± 0.20	0.11 ± 0.00
Total MDAs	1.74 ± 0.47	5.03 ± 0.22	0.10 ± 0.07	0.21 ± 0.01	0.01 ± 0.01	0.00 ± 0.00	**** 0.01 ± 0.01	0.02 ± 0.00	0.21 ± 0.09	0.11 ± 0.00	0.84 ± 0.53	0.29 ± 0.01
Total toxic alkaloids	** 12.63 ± 10.97	5.03 ± 0.22	**** 1.48 ± 0.51	0.21 ± 0.01	* 0.09 ± 0.14	0.00 ± 0.00	N.D.	0.02 ± 0.00	**** 4.13 ± 1.56	0.11 ± 0.00	**** 27.57 ± 20.31	0.29 ± 0.01

* *p* < 0.05, ** *p* < 0.01, **** *p* < 0.0001 compared with corresponding Paofupian group; N.D.: not detectable, below the listed LLOQ of each rat tissue in Table A1.

**Table 3:** Comparison of dose-normalized toxic alkaloids content (× 10^−3^) in rat plasma, urine and different organs between multiple dose of 30 g/kg Heishunpian and multiple dose of 30 g/kg Paofupian to rats. (*n* = 8).

Tissue	Liver		Kidney		Heart		Brain		Plasma		Urine	
Compound	Heishunpian	Paofupian	Heishunpian	Paofupian	Heishunpian	Paofupian	Heishunpian	Paofupian	Heishunpian	Paofupian	Heishunpian	Paofupian
AC	N.D.	N.D.	N.D.	N.D.	N.D.	N.D.	N.D.	N.D.	N.D.	N.D.	N.D.	N.D.
HA	N.D.	N.D.	N.D.	N.D.	N.D.	N.D.	N.D.	N.D.	N.D.	N.D.	102.96 ± 46.64	N.D.
MA	N.D.	N.D.	N.D.	N.D.	N.D.	N.D.	N.D.	N.D.	N.D.	N.D.	N.D.	N.D.
Total DDAs	N.D.	N.D.	N.D.	N.D.	N.D.	N.D.	N.D.	N.D.	N.D.	N.D.	102.96 ± 46.64	N.D.
BAC	3.01 ± 1.51	4.23 ± 0.28	0.51 ± 0.11	0.30 ± 0.02	0.13 ± 0.02	0.11 ± 0.01	N.D.	N.D.	0.16 ± 0.05	0.14 ± 0.01	12.18 ± 8.84	3.02 ± 0.20
BHA	** 2.82 ± 0.83	0.78 ± 0.05	**** 1.04 ± 0.20	0.54 ± 0.04	**** 0.17 ± 0.03	0.26 ± 0.02	N.D.	N.D.	0.16 ± 0.06	0.25 ± 0.02	14.38 ± 8.43	6.74 ± 0.45
BMA	*** 2.87 ± 0.97	0.81 ± 0.05	0.49 ± 0.11	0.35 ± 0.02	0.11 ± 0.03	0.09 ± 0.01	**** 0.05 ± 0.01	0.03 ± 0.00	0.09 ± 0.05	0.03 ± 0.00	8.06 ± 7.33	2.84 ± 0.19
Total MDAs	**** 8.70 ± 2.52	5.82 ± 0.39	**** 2.05 ± 0.40	1.21 ± 0.08	** 0.41 ± 0.04	0.45 ± 0.03	**** 0.05 ± 0.01	0.03 ± 0.00	0.41 ± 0.10	0.38 ± 0.15	34.62 ± 23.87	12.60 ± 0.85
Total toxic alkaloids	**** 8.70 ± 2.52	5.82 ± 0.39	**** 2.05 ± 0.40	1.21 ± 0.08	** 0.41 ± 0.04	0.45 ± 0.03	**** 0.05 ± 0.01	0.03 ± 0.00	0.41 ± 0.10	0.38 ± 0.15	**** 137.58 ± 49.70	12.60 ± 0.85

** *p* < 0.01, *** *p* < 0.001, **** *p* < 0.0001 compared with corresponding Paofupian group; N.D.: not detectable, below the listed LLOQ of each rat tissue in Table A1.

## Appendix A

**Table A1:** Lower Limit of Quantification (LLOQ) and linearity of AC, HA, MA, BAC, BHA and BMA in rat heart, liver, brain, urine, kidney and plasma by the current developed LC/MS/MS method.

Tissue	Heart			Liver					Brain				Urine				Kidney				Plasma			
Compound	LLOQ ng/mL	RSD %	RE %	Linear Range ng/mL	LLOQ ng/mL	RSD %	RE %	Linear Range ng/mL	LLOQ ng/mL	RSD %	RE %	Linear Range ng/mL	LLOQ ng/mL	RSD %	RE %	Linear Range ng/mL	LLOQ ng/mL	RSD %	RE %	Linear Range ng/mL	LLOQ ng/mL	RSD %	RE %	Linear Range ng/mL
AC	1	16.24	−2.06	1–20	2	5.12	−0.41	2–20	2	3.05	2.35	2–100	2	3.88	5.4	1–200	1	1.46	1.49	1–50	0.5	4.74	16.16	0.5–100
HA	1	12.14	−2.93	1–20	1	1.7	6.19	1–20	2	7.81	0.39	2–100	0.5	6.87	7.27	0.5–200	0.5	2.66	2.44	0.5–50	0.5	7.30	5.84	0.5–100
MA	1	8.32	0.01	1–20	1	1.74	17.21	1–20	2	7.53	0.23	2–100	0.5	9.48	0.47	0.5–200	0.5	12.03	−8.39	0.5–50	0.5	9.89	5.79	0.5–100
BAC	1	13.46	0.11	1–20	1	6.42	9.14	1–20	2	5.87	−7.78	2–100	0.5	1.67	−2.25	0.5–200	0.5	9.32	2.67	0.5–50	0.5	5.72	5.89	0.5–100
BHA	1	2.27	7.05	1–20	1	3.89	10.02	1–20	2	10.18	−5.5	2–100	0.5	5.1	−12.24	0.5–200	0.5	17.27	1.1	0.5–50	0.5	1.18	−1.64	0.5–100
BMA	1	4.99	11.42	1–20	1	5.39	2.83	1–20	2	11.83	−1.03	2–100	2	3.13	−4.32	2–200	0.5	10.62	−0.05	0.5–50	0.5	1.22	5.52	0.5–100

**Table A2:** Summary of accuracy, precision, recovery, matrix effect, and stability information of AC, HA, MA, BAC, BHA and BMA in rat heart homogenate (*n* = 5).

Analytes	Concentration (ng/mL)	Intra-Day (%, RSD)	Inter-Day (%, RSD)	Accuracy (%, RE)	Absolute Recovery (%, Mean ± SD)	Matrix Effect (%, RSD)	Stability (%, Mean ± SD)			
							4 h at Room Temperature	12 h at 8 °C	3 Freeze-Thaw Cycles	30 Days at –80 °C
AC	2	11.21	5.32	2.11	48.83 ± 11.47	10.04	66.64 ± 12.85	71.12 ± 9.60	105.41 ± 12.97	69.46 ± 0.48
	5	0.04	1.55	0.03	36.19 ± 4.35	3.81	N.A.	N.A.	N.A.	N.A.
	10	0.08	3.99	0.95	35.58 ± 1.53	8.72	88.50 ± 10.76	72.77 ± 7.56	109.17 ± 4.33	129.82 ± 5.89
HA	2	11.25	1.31	−11.08	52.47 ± 4.89	11.25	80.32 ± 4.42	97.99 ± 14.73	101.72 ± 14.32	94.47 ± 6.18
	5	6.54	4.67	2.95	48.65 ± 2.96	6.54	N.A.	N.A.	N.A.	N.A.
	10	10.52	8.86	−13.62	34.06 ± 1.17	9.89	92.43 ± 9.29	75.79 ± 9.84	107.78 ± 7.09	127.00 ± 5.00
MA	2	8.71	6.65	−3.11	27.08 ± 3.41	9.26	68.43 ± 3.93	90.60 ± 13.11	98.31 ± 7.88	103.54 ± 1.54
	5	9.37	7.02	0.78	22.02 ± 0.91	8.86	N.A.	N.A.	N.A.	N.A.
	10	10.92	2.07	−1.19	15.93 ± 0.10	11.42	82.54 ± 9.02	80.64 ± 8.44	94.76 ± 1.83	125.96 ± 4.38
BAC	2	11.06	3.06	1.76	55.06 ± 9.84	11.06	104.47 ± 10.69	146.84 ± 11.79	86.81 ± 5.92	86.34 ± 6.75
	5	7.74	5.39	–6.22	36.39 ± 4.63	7.74	N.A.	N.A.	N.A.	N.A.
	10	5.17	6.85	2.49	29.75 ± 2.12	5.17	115.46 ± 6.90	157.93 ± 13.13	111.46 ± 7.11	105.17 ± 2.94
BHA	2	7.2	7.3	1.22	38.20 ± 7.50	7.2	102.36 ± 3.86	146.72 ± 5.14	94.32 ± 11.40	89.70 ± 2.61
	5	9.19	7.8	9.41	25.50 ± 2.69	9.19	N.A.	N.A.	N.A.	N.A.
	10	6.24	3.76	2.16	19.91 ± 1.68	6.24	86.60 ± 10.07	131.51 ± 3.44	97.57 ± 6.87	105.87 ± 1.33
BMA	2	5.56	7.73	−10.38	59.61 ± 4.38	5.14	92.84 ± 5.27	185.65 ± 14.98	118.67 ± 6.02	69.01 ± 4.47
	5	11	6.58	10.14	60.62 ± 1.69	13.23	N.A.	N.A.	N.A.	N.A.
	10	6.59	2.29	−0.44	51.37 ± 2.95	6.59	78.04 ± 5.01	113.08 ± 5.77	73.44 ± 4.82	139.14 ± 10.84

N.A.: No requirements for stability test on analytes’ Middle of Quantification (MOQs) in U.S. FDA [28] Guidelines.

**Table A3:** Summary of accuracy, precision, recovery, matrix effect and stability information of AC, HA, MA, BAC, BHA and BMA in rat liver homogenate (*n* = 5).

Analytes	Concentration (ng/mL)	Intra-Day (%, RSD)	Inter-Day (%, RSD)	Accuracy (%, RE)	Absolute Recovery (%, Mean ± SD)	Matrix Effect (%, RSD)	Stability (%, Mean ± SD)			
							4 h at Room Temperature	12 h at 8 °C	3 Freeze-Thaw Cycles	30 Days at −80 °C
AC	5	10.83	9.21	−0.74	16.27 ± 1.76	10.83	109.37 ± 9.10	95.40 ± 4.17	119.10 ± 11.54	123.35 ± 14.20
	10	4.2	2.04	2.89	15.44 ± 0.65	4.2	N.A.	N.A.	N.A.	N.A.
	50	1.8	0.06	0.6	16.85 ± 0.30	1.8	88.31 ± 5.74	99.15 ± 2.97	91.81 ± 2.92	98.03 ± 14.28
HA	5	6.17	5.05	1.36	17.48 ± 1.08	6.17	101.74 ± 2.56	91.27 ± 6.35	108.72 ± 3.18	89.69 ± 4.36
	10	7.85	0.08	0.07	17.00 ± 1.33	7.85	N.A.	N.A.	N.A.	N.A.
	50	1.75	0.06	−3.88	18.35 ± 0.32	1.75	106.79 ± 1.71	95.52 ± 2.14	110.29 ± 4.89	95.45 ± 13.53
MA	5	6.08	3	−2.47	16.46 ± 1.00	6.08	101.76 ± 0.57	93.24 ± 4.46	120.36 ± 6.72	92.81 ± 10.90
	10	2.75	8.07	-0.01	15.78 ± 0.43	2.75	N.A.	N.A.	N.A.	N.A.
	50	3.52	0.05	0.14	16.94 ± 0.60	3.52	94.11 ± 4.29	96.41 ± 3.02	99.94 ± 4.80	92.27 ± 12.05
BAC	5	9.37	2.81	−1.07	2.64 ± 0.25	9.37	138.93 ± 7.85	85.36 ± 8.33	141.29 ± 11.86	127.23 ± 9.12
	10	8.85	0.53	2.23	3.38 ± 0.30	8.85	N.A.	N.A.	N.A.	N.A.
	50	2.65	3.04	−0.23	2.96 ± 0.08	2.65	97.68 ± 5.58	97.81 ± 4.03	100.15 ± 4.94	106.27 ± 6.59
BHA	5	5.69	6.67	9.08	2.60 ± 0.15	5.69	141.14 ± 9.36	93.21 ± 7.16	161.20 ± 10.89	137.34 ± 12.78
	10	11.98	5.63	−0.17	4.22 ± 0.51	11.98	N.A.	N.A.	N.A.	N.A.
	50	3.61	3.99	−5.59	3.12 ± 0.11	3.61	106.85 ± 3.50	95.41 ± 2.42	112.54 ± 3.40	140.18 ± 14.86
BMA	5	5.51	2.22	6.12	2.47 ± 0.14	5.51	104.19 ± 9.20	87.37 ± 3.96	113.15 ± 9.93	100.35 ± 11.19
	10	5.84	4.62	−3.09	2.71 ± 0.16	5.84	N.A.	N.A.	N.A.	N.A.
	50	2.78	5.54	−10.32	2.36 ± 0.07	2.78	106.43 ± 5.48	93.28 ± 4.63	93.12 ± 0.68	86.70 ± 6.88

N.A.: No requirements for stability test on analytes’ Middle of Quantification (MOQs) in U.S. FDA [28] Guidelines.

**Table A4:** Summary of accuracy, precision, recovery, matrix effect and stability information of AC, HA, MA, BAC, BHA and BMA in rat brain homogenate (*n* = 5).

Analytes	Concentration (ng/mL)	Intra-Day (%, RSD)	Inter-Day (%, RSD)	Accuracy (%, RE)	Absolute Recovery (%, Mean ± SD)	Matrix Effect (%, RSD)	Stability (%, Mean ± SD)			
							4 h at Room Temperature	12 h at 8 °C	3 Freeze-Thaw Cycles	30 Days at −80 °C
AC	2	5.55	15.2	−1.88	27.48 ± 1.53	5.55	95.56 ± 10.84	100.24 ± 12.29	105.91 ± 5.19	114.35 ± 9.72
	5	7.87	6.76	−1.72	34.63 ± 2.73	7.87	N.A.	N.A.	N.A.	N.A.
	10	8.97	3.06	−1.17	32.03 ± 2.87	8.97	98.68 ± 6.63	105.49 ± 7.80	101.30 ± 4.77	111.57 ± 11.56
HA	2	4.35	15.2	0.17	33.56 ± 1.46	4.35	93.24 ± 5.68	100.36 ± 7.15	98.89 ± 3.59	97.16 ± 6.95
	5	2.9	2.02	−4.82	31.15 ± 0.90	2.9	N.A.	N.A.	N.A.	N.A.
	10	4.34	1.51	3.29	33.46 ± 1.45	4.34	104.40 ± 3.42	104.03 ± 5.74	101.13 ± 4.34	104.66 ± 8.08
MA	2	3.82	15.19	0.42	26.02 ± 0.99	3.82	94.97 ± 8.67	100.26 ± 9.16	100.46 ± 0.77	90.20 ± 10.51
	5	3.2	1.59	−13.39	23.74 ± 0.76	3.2	N.A.	N.A.	N.A.	N.A.
	10	4.65	3.26	−0.49	25.60 ± 1.19	4.65	100.93 ± 5.61	104.19 ± 6.67	101.38 ± 2.04	93.22 ± 7.50
BAC	2	3.38	18.14	−0.37	4.80 ± 0.16	3.38	109.55 ± 14.53	100.56 ± 6.29	105.67 ± 18.95	225.25 ± 1.44
	5	9.9	6.8	−8.87	4.46 ± 0.44	9.9	N.A.	N.A.	N.A.	N.A.
	10	4.08	6.48	−4.88	5.78 ± 0.24	4.08	103.91 ± 5.44	96.30 ± 2.65	90.73 ± 6.52	184.82 ± 11.92
BHA	2	3.22	7.16	3.48	5.21 ± 0.17	3.22	89.92 ± 7.02	95.97 ± 8.77	86.65 ± 10.79	166.17 ± 14.83
	5	6.26	4.47	−13.7	3.65 ± 0.23	6.26	N.A.	N.A.	N.A.	N.A.
	10	4.5	4.28	−1.36	7.42 ± 0.33	4.5	101.38 ± 1.24	100.01 ± 6.63	88.47 ± 3.88	1&7.87 ± 4.76
BMA	2	9.48	15.56	−1.38	4.15 ± 0.39	9.48	144.55 ± 14.24	94.36 ± 16.14	98.54 ± 6.93	229.74 ± 7.66
	5	3.12	5.88	−2.41	3.97 ± 0.12	3.12	N.A.	N.A.	N.A.	N.A.
	10	3.52	2.54	−3.12	4.74 ± 0.17	3.52	83.7 ± 2.74	105.89 ± 5.97	83.46 ± 5.53	136.53 ± 5.32

N.A.: No requirements for stability test on analytes’ Middle of Quantification (MOQs) in U.S. FDA [28] Guidelines.

**Table A5:** Summary of accuracy, precision, recovery, matrix effect and stability information of AC, HA, MA, BAC, BHA and BMA in rat urine (*n* = 5).

Analytes	Concentration (ng/mL)	Intra-Day (%, RSD)	Inter-Day (%, RSD)	Accuracy (%, RE)	Absolute Recovery (%, Mean ± SD)	Matrix Effect (%, RSD)	Stability (%, Mean ± SD)			
							4 h at Room Temperature	12 h at 8 °C	3 Freeze-Thaw Cycles	30 Days at −80 °C
AC	2	3.88	4.67	5.4	84.41 ± 4.15	3.88	87.05 ± 13.58	126.77 ± 4.77	79.91 ± 12.50	73.12 ± 7.69
	10	10.24	11.5	−2.94	93.28 ± 5.62	10.24	N.A.	N.A.	N.A.	N.A.
	50	2.7	1.87	0.31	98.79 ± 3.53	2.7	117.59 ± 8.17	101.92 ± 6.69	115.14 ± 5.79	109.04 ± 3.36
HA	2	6.11	3.87	3.15	100.06 ± 1.70	6.11	76.89 ± 5.39	95.39 ± 4.69	84.95 ± 1.67	88.36 ± 2.51
	10	6.21	7.69	−10.93	94.11 ± 1.80	6.21	N.A.	N.A.	N.A.	N.A.
	50	3.98	1.62	1.63	101.24 ± 3.59	3.98	113.66 ± 2.41	98.69 ± 1.66	129.99 ± 1.85	107.34 ± 0.85
MA	2	3.5	5.74	9.99	96.95 ± 3.15	3.5	80.31 ± 1.09	97.99 ± 4.14	81.96 ± 0.65	82.86 ± 1.14
	10	4.12	6.04	−7.28	92.25 ± 2.45	4.12	N.A.	N.A.	N.A.	N.A.
	50	6.59	3.77	0.46	98.47 ± 3.07	6.59	111.56 ± 0.51	98.19 ± 2.31	119.99 ± 1.65	103.40 ± 3.53
BAC	2	3.73	6.12	9.14	106.46 ± 2.39	3.73	89.96 ± 13.19	100.22 ± 10.17	85.13 ± 11.07	81.30 ± 6.12
	10	7.11	3	0.47	99.19 ± 0.80	7.11	N.A.	N.A.	N.A.	N.A.
	50	3.3	11.7	2.59	103.41 ± 1.48	3.3	102.55 ± 2.13	122.66 ± 4.15	111.31 ± 0.85	105.31 ± 3.33
BHA	2	3.81	8.28	5.11	98.27 ± 2.68	3.81	76.31 ± 8.32	99.16 ± 6.30	94.79 ± 3.84	96.08 ± 4.23
	10	5.89	4.05	−1.71	96.69 ± 1.86	5.89	N.A.	N.A.	N.A.	N.A.
	50	7.29	6.7	−1.64	100.71 ± 2.40	7.29	96.92 ± 1.62	116.89 ± 2.81	135.02 ± 1.75	101.46 ± 2.66
BMA	10	4.99	7.41	13.21	100.05 ± 2.61	4.99	83.77 ± 4.06	103.23 ± 4.57	92.87 ± 11.00	21.21 ± 3.08
	50	8.53	8.53	0.27	104.94 ± 1.96	8.53	N.A.	N.A.	N.A.	N.A.
	100	6.52	6.52	3.07	110.31 ± 3.53	6.52	109.94 ± 1.16	126.82 ± 1.82	103.55 ± 5.31	90.84 ± 0.72

N.A.: No requirements for stability test on analytes’ Middle of Quantification (MOQs) in U.S. FDA [28] Guidelines.

**Table A6:** Summary of accuracy, precision, recovery, matrix effect and stability information of AC, HA, MA, BAC, BHA and BMA in rat plasma (*n* = 5).

Analytes	Concentration (ng/mL)	Intra-Day (%, RSD)	Inter-Day (%, RSD)	Accuracy (%, RE)	Absolute Recovery (%, Mean ± SD)	Matrix Effect (%, RSD)	Stability (%, Mean ± SD)			
							4 h at Room Temperature	12 h at 8 °C	3 Freeze-Thaw Cycles	30 Days at –80 °C
AC	2	6.61	5.34	−2.34	71.28 ± 4.71	6.61	101.30 ± 7.24	100.54 ± 0.29	112.76 ± 9.08	164.14±8.87
	5	3.98	8.03	-6.10	67.46 ± 2.69	3.98	N.A.	N.A.	N.A.	N.A.
	20	5.79	3.74	5.20	82.80 ± 4.80	5.79	100.21 ± 3.55	88.89 ± 4.26	108.61 ± 3.01	108.22 ± 0.03
HA	2	3.23	5.56	−9.89	67.00 ± 2.16	3.23	125.20 ± 7.65	100.01 ± 3.38	119.58 ± 1.07	118.18±7.50
	5	2.19	8.19	−14.04	59.65 ± 0.53	0.88	N.A.	N.A.	N.A.	N.A.
	20	4.03	6.06	−1.04	69.54 ± 2.80	4.03	133.03 ± 4.53	98.81 ± 4.70	121.92 ± 5.95	114.49 ± 2.00
MA	2	5.31	5.57	−13.57	71.34 ± 3.79	5.31	118.52 ± 11.80	80.92 ± 3.75	103.79 ± 1.63	99.84 ± 5.24
	5	2.00	2.05	−6.56	88.82 ± 1.78	2.00	N.A.	N.A.	N.A.	N.A.
	20	3.74	5.04	8.29	92.04 ± 3.45	3.74	115.71 ± 4.75	87.06 ± 4.38	106.95 ± 1.82	98.85 ± 0.90
BAC	2	3.24	4.98	−3.11	82.85 ± 2.67	3.23	114.26 ± 6.75	113.52±9.71	110.02 ± 5.14	79.65 ± 3.17
	5	3.63	7.28	2.41	111.31 ± 4.04	3.63	N.A.	N.A.	N.A.	N.A.
	20	2.55	3.27	−8.37	90.32 ± 2.31	2.55	123.28 ± 5.74	95.37 ± 0.09	114.72 ± 2.84	103.64 ± 2.26
BHA	2	7.46	7.65	9.60	80.58 ± 6.01	7.46	111.19 ± 14.68	94.49 ± 3.94	108.49 ± 3.62	98.50 ± 5.31
	5	4.72	8.91	−2.83	95.82 ± 4.52	4.72	N.A.	N.A.	N.A.	N.A.
	20	4.77	5.51	−6.23	87.84 ± 4.19	4.77	122.77 ± 4.71	89.36 ± 1.46	112.25 ± 3.59	102.16 ± 2.74
BMA	2	5.25	14.89	8.22	68.49 ± 1.99	2.91	109.38 ± 4.75	132.63 ± 9.45	128.71 ± 14.56	48.11 ± 7.51
	5	9.42	12.40	5.92	100.65 ± 10.12	10.06	N.A.	N.A.	N.A.	N.A.
	20	2.52	2.15	−13.54	94.23 ± 2.39	2.91	117.83 ± 5.66	95.58 ± 1.79	106.33 ± 5.24	98.74 ± 8.44

N.A.: No requirements for stability test on analytes’ Middle of Quantification (MOQs) in U.S. FDA [28] Guidelines.

**Table A7:** Summary of accuracy, precision, recovery, matrix effect and stability information of AC, HA, MA, BAC, BHA and BMA in rat kidney homogenate (*n* = 5).

Analytes	Concentration (ng/mL)	Intra-Day (%, RSD)	Inter-Day (%, RSD)	Accuracy (%, RE)	Absolute Recovery (%, Mean ± SD)	Matrix Effect (%, SD)	Stability (%, Mean ± SD)			
							4 h at Room Temperature	12 h at 8 °C	3 Freeze-Thaw Cycles	30 Days at −80 °C
AC	1	1.46	11.68	1.49	90.67 ± 9.23	3.20	102.31 ± 3.11	53.29 ± 5.38	106.24 ± 8.01	89.62 ± 7.12
	5	10.43	10.43	5.16	79.64 ± 2.48	0.45	N.A.	N.A.	N.A.	N.A.
	20	6.53	6.53	4.7	81.40 ± 10.34	1.85	63.75 ± 5.51	44.14 ± 5.58	75.04 ± 6.90	85.86 ± 5.49
HA	1	5.08	10.02	5.77	76.99 ± 8.35	1.60	53.22 ± 2.99	70.71 ± 0.52	47.33 ± 3.15	88.69 ± 8.19
	5	5.22	10.64	−1.03	79.36 ± 6.23	1.07	N.A.	N.A.	N.A.	N.A.
	20	0.46	1.38	0.64	92.53 ± 12.25	2.07	65.23 ± 3.45	82.41 ± 1.36	38.14 ± 1.10	87.13 ± 1.33
MA	1	5.28	10.12	8.75	87.61 ± 5.49	0.95	74.54 ± 1.05	47.25 ± 5.35	92.18 ± 5.12	98.80 ± 5.34
	5	4.78	11.87	−4.37	78.46 ± 5.63	0.87	N.A.	N.A.	N.A.	N.A.
	20	2.18	1.77	1.86	98.72 ± 12.45	1.74	68.62 ± 2.81	34.29 ± 2.14	84.91 ± 0.92	107.02 ± 2.69
BAC	1	9.8	12.28	1.64	101.51 ± 10.51	1.12	106.14 ± 5.48	49.46 ± 4.12	120.73 ± 8.11	103.41 ± 5.29
	5	7.15	7.47	−3.42	91.28 ± 14.16	1.26	N.A.	N.A.	N.A.	N.A.
	20	8.65	1.46	0.91	86.44 ± 13.89	1.21	108.06 ± 12.04	56.57 ± 3.82	127.04 ± 4.56	87.07 ± 5.42
BHA	1	6.11	6.53	1.63	96.88 ± 14.86	1.66	93.91 ± 13.38	63.66 ± 10.36	98.44 ± 13.02	108.47 ± 2.67
	5	9.47	4.83	0.91	72.70 ± 9.55	0.98	N.A.	N.A.	N.A.	N.A.
	20	6.29	1.76	1.32	87.82 ± 14.79	1.33	108.66 ± 2.18	46.05 ± 6.01	120.96 ± 2.55	92.94 ± 3.62
BMA	1	10.16	8.06	−2.43	93.99 ± 11.26	1.75	79.79 ± 1.43	77.59 ± 9.88	96.94 ± 10.13	97.18 ± 7.90
	5	7.74	3.58	3.52	78.27 ± 8.32	0.64	N.A.	N.A.	N.A.	N.A.
	20	3.43	6.06	2.84	76.90 ± 13.40	1.01	97.12 ± 7.38	49.49 ± 10.83	112.43 ± 2.61	82.85 ± 1.04

N.A.: No requirements for stability test on analytes’ Middle of Quantification (MOQs) in U.S. FDA [28] Guidelines.
